# Neutral glycans from sandfish skin can reduce friction of polymers

**DOI:** 10.1098/rsif.2016.0103

**Published:** 2016-03

**Authors:** Boštjan Vihar, Franz Georg Hanisch, Werner Baumgartner

**Affiliations:** 1 Institute of Biomedical Mechatronics, Johannes Kepler University of Linz, Altenbergerstrasse 69, 4040 Linz, Austria; 2 Institute for Biology II, RWTH Aachen University, Worringerweg 3, 52056 Aachen, Germany; 3 Institute for Biochemistry II, University of Cologne, Joseph-Stelzmann-Strasse 52, 50931 Cologne, Germany

**Keywords:** *Scincus*, integument, mass spectroscopy, mannose, sand swimming

## Abstract

The lizard *Scincus scincus*, also known as sandfish, can move through aeolian desert sand in a swimming-like manner. A prerequisite for this ability is a special integument, i.e. scales with a very low friction for sand and a high abrasion resistance. Glycans in the scales are causally related to the low friction. Here, we analysed the glycans and found that neutral glycans with five to nine mannose residues are important. If these glycans were covalently bound to acrylic polymers like poly(methyl methacrylate) or acrylic car coatings at a density of approximately one molecule per 4 nm², friction for and adhesion of sand particles could be reduced to levels close to those observed with sandfish scales. This was also found true, if the glycans were isolated from sources other than sandfish scales like plants such as almonds or mistletoe. We speculate that these neutral glycans act as low density spacers separating sand particles from the dense scales thereby reducing van der Waals forces.

## 1. Introduction

The scincid lizard genus *Scincus* [1], a group within the order of Squamata, is distributed over the huge desert belt ranging from the African west coast (Morocco to Senegal) through the Sahara and the Arabian Peninsula into Jordan, Iraq and southwest Iran [1–3]. The sandfish *Scincus scincus* is known for its ability to move through desert sand very fast, as if it was swimming [1–7]. This is in contrast to other desert reptiles which can bury themselves in the sand to hide from predators, but do not move in the sand over significant distances. It was found that the sandfish exhibits an in-plane meandering motion and reaches velocities of up to 300 mm s^−1^ which corresponds to 1.5 times the full-body length per second [6,7] when completely submerged in sand.

**Figure 1.**
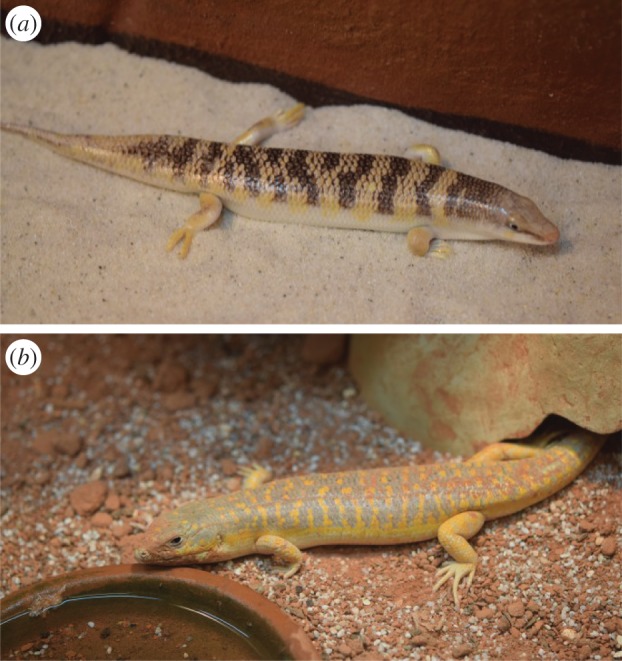
Comparison of the appearance of the sandfish *Scincus scincus* and the Berber skink *Eumeces schneideri*. (*a*) An adult specimen of *S. scincus* photographed during a short stay on the sand surface in a terrarium. As can be seen clearly, the scales are not scratched as they reflect brightly and no sand adheres onto the surface, although the animal just rose up from the sand. (*b*) The genetically closely related but not sand swimming *E. schneideri* is shown for comparison. These lizards tend to dig in the sandy ground, but do not actively bury. Clearly, ground particles adhere onto the scales, especially at the snout and the legs, the body parts which are mainly used to dig.

Life underneath loose, aeolian sand requires morphological key adaptations, such as a shovel-shaped snout with the lower jaw wedged into the upper jaw, reduced ear openings and nostrils, a subquadrangular cross section of the body, and strongly developed limbs with fringed digits and toes (figure 1*a*). These adaptations were well known already to early authors [1–3]. However, most adaptations to life underneath the sharp-edged sand are poorly understood. Especially, their skin shows a high resistance against abrasion together with a low friction angle to sand in comparison with other materials [4,5,8,9]. The sandfish's skin exhibits a friction angle of only *θ* = 19–21° (corresponding friction coefficient *μ* = tan *θ* = 0.32–0.37), whereas the friction angle of steel is *θ* = 25° (*μ* = 0.47), that of Teflon is *θ* = 35° (*μ* = 0.70) and the epidermis of the closely related but not sand swimming lizard *Scincopus fasciatus θ* = 36° (*μ* = 0.72) or *Eumeces schneideri θ* = 37° (*μ* = 0.74) [5,8]. This low friction allows an energy saving, swimming-like movement through sand. Furthermore, the skin shows extraordinary resilience to abrasion. It can even withstand mild sand blasting, which mechanically stronger materials cannot [6,7]. Biochemical analyses have shown that the epidermis mainly consists of β-keratins (corneous beta proteins [8]) which are strongly glycosylated [9,10]. Besides friction and abrasion resistance the scales exhibit a very low adhesion to (sand) particles due to van der Waals forces [9]. In recent works, we could show that these glycans are causally involved in the friction reduction by reducing adhesion of particles with the scales [9]. This can be seen easily when comparing the sandfish (figure 1*a*) to a closely related scincid lizard, the Berber skink *E. schneideri* (figure 1*b*) which does not swim in sand. While the scales of *S. scincus* are immediately clean, bright and unscratched when emerging from the sand, the scales of *E. schneideri* are often covered with sand or dirt particles adhering to the surface.

Previous studies have shown that if covalently bound to glass, the glycans purified from sandfish scales efficiently reduce adhesion of sand on this surface. Also the friction of sand on the modified glass is reduced. [9]. It was found by use of lectins, i.e. proteins that specifically bind to specific glycans, that different glycans are present on the keratins forming the sandfish scales. It was found that *O*- as well as *N*-linked glycans can be detected, complex, mannose-rich species as well as sialic acid-rich carbohydrates.

The purpose of the following investigation was to determine which glycan structures are responsible for the adhesion (and friction) reduction. Furthermore, we used the identified glycans to modify other materials such as thermoplastic polymers to see if the natural glycans can reduce adhesion and friction of artificial materials, which are of interest for engineering. Finally, we tried to shed more light on how the glycans can reduce adhesion and friction on a molecular basis.

## 2. Material and methods

### 2.1. Animals

The lizards of the species *S. scincus* as well as *E. schneideri* (shown here for comparison) were kept in private terraria and the owners of these lizards donated the shed exuviae from moulting to our institute for investigations. These exuviae were found during routine cleaning of a terrarium. No experiments were performed on living species or on samples obtained from living species or individuals killed for this purpose.

### 2.2. Enzymatic cleavage and methylation of *N*-glycans for mass spectroscopy

Cleaving was performed similar to the methods described by Staudt *et al*. [9]. Dried keratins were dissolved in 100 µl 50 mM NH_4_HCO_3_ and submitted to tryptic digestion at 37°C for approximately 16 h. Trypsin was inactivated at 95°C for 5 min. Digested sample was dried by vacuum centrifugation and solubilized in 50 µl 50 mM NH_4_HCO_3_. To cleave the *N*-linked glycans from the peptide an aliquot of 0.5 µl peptide-*N*-glycosidase F, shortly named PNGase F (NEB, Frankfurt, Germany), was added and *N*-deglycosylation was performed at 37°C for at least 16 h. Liberated *N*-glycans were separated from peptides by solid-phase extraction on C18-cartridges (Agilent Technologies, Waldbronn, Germany). *N*-glycans in the flow through were dried by vacuum rotation and in a desiccator for 1 h in the presence of P_2_O_5_/KOH. Methylation was performed in 100 µl water-free dimethylsulfoxide containing finely dispersed NaOH (30 min at 22°C), followed by addition of 50 µl methyliodide (30 min at 22°C). After methylation, 300 µl chloroform was added, and the sample was repeatedly extracted with 200 µl water. The chloroform phase was dried under N_2_ and methylated glycans were dissolved in 20 µl methanol.

### 2.3. Tandem matrix-assisted laser desorption ionization time of flight mass spectrometry

Matrix-assisted laser desorption ionization (MALDI) mass spectrometry was performed on an UltrafleXtreme instrument (Bruker Daltonics). The permethylated glycans (approx. 500 ng) contained in methanol were applied to the stainless steel target by mixing a 0.5 µl aliquot of sample with 1.0 µl of matrix (saturated solution of 2,5-dihydroxybenzoic acid in ACN/0.1% TFA, 1:2). Alternatively, 0.75 µl of sample was mixed with 0.75 µl α-cyano-4-hydroxycinnamic acid (Bruker, Bremen, Germany) matrix solution on the MALDI target and subjected to mass spectrometric analysis as described by Breloy *et al*. [11].

### 2.4. *N*-Glycan extraction for polymer modification

Dissolved proteins [5,9] were dialysed to phosphate-buffered saline and to 45 µl of the solution, 1 µl of 5% sodium dodecylsulfate and 1.5 µl of 1.67 M 2-mercaptoethanol were added. The mix was vortexed and incubated at 37°C for 20 min. In total, 5 µl of Nonidet NP40 was added with addition of 4 µl of PNGase F (*N*-glycosidase) and the incubation was continued overnight at 37°C. To extract the liberated glycans, 150 µl of ice-cold 100% ethanol were added and the mix was gently stirred for 20 min at 0°C. This was followed by a 5 min centrifugation at 12 000*g*. The supernatant, containing glycans and mercaptoethanol, was transferred to a fresh vial and dried by vacuum rotation.

For later experiments, the glycans were further prepared, using a simple anion-exchange chromatography protocol with a digital pipette (Rainin, Mettler Toledo, Columbus, OH, USA). Using a strong anion-exchange resin and the preinstalled protocol, acidic and other negatively charged glycans were separated from neutral (such as high-mannose) glycans.

### 2.5. Coating of poly(methyl methacrylate) and GW34

Owing to its wide applicability [12,13], and its adaptable acrylic group, shared also by other materials, poly(methyl methacrylate) (PMMA) was an interesting candidate for covalently binding glycans to the polymer surface.

The coating process took place in two stages. The first step was creating a free primary amine on the PMMA surface, using an amino linker. This was previously achieved by different methods, using either organic solvents [14] or boric acid at high pH values [15]. A new protocol was devised, based on those studies, using a 10% H_2_N(CH_2_)_6_NH_2_ (hexamethylene diamine) solution in 2-propanol. PMMA foil was soaked in the solution for 20 min at room temperature.

**Figure 2.**
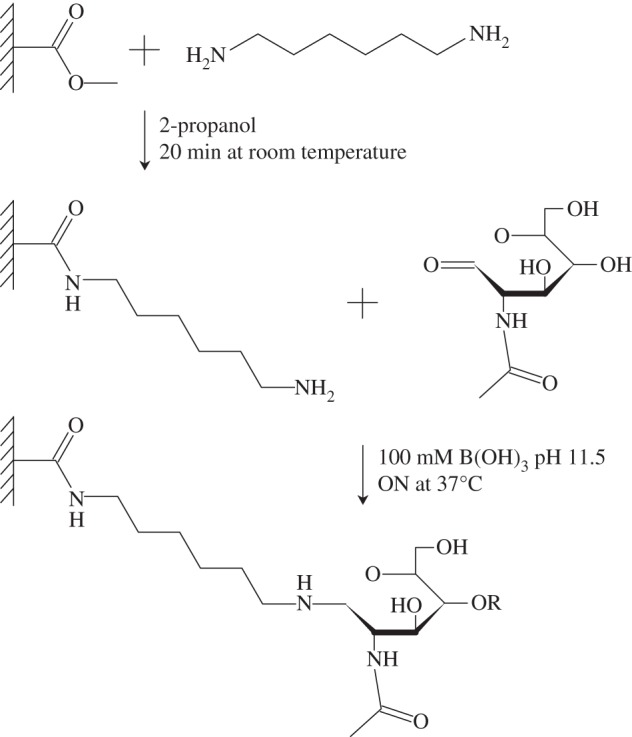
The proposed mechanism of PMMA glycosylation. In our method, first the amino-linker hexamethylene diamine is attached to the acrylic surface by attacking the methoxy group, creating a free primary amine on the surface. In the second step, a glycan is linked to the amine through a reductive amination reaction. For this to work, the first GlcNAc has to undergo a ring-opening, thus creating a free aldehyde group, which can react with the free primary amine on the acrylic surface.

The second step was binding the glycans, using a reductive amination reaction, between a primary amine and a ketone group of the GlcNAc. This was performed, as suggested by the study of Bulmus *et al*. [16]. The amine-treated PMMA samples were exposed to the glycans in a 100 mM solution of B(OH)_3_/NaOH pH 11.5 and incubated overnight at 37°C. The success of surface modification was determined indirectly by contact angle measurement, friction, adhesion force measurements and surface density determination. A direct definitive proof of the chemical mechanism behind the modification could not be established; however, we propose the mechanism shown in figure 2.

### 2.6. Surface density determination

To determine the surface density of bound glycans, an indirect approach was necessary. PMMA plates (25 × 60 mm) were activated with the amino-linker hexamethylene diamine as described above. Then an aminoreactive fluorescein isothiocyanate (FITC-NHS, Sigma) at a concentration of 100 µM ml^−1^ was coupled according to the protocol for glycan bonding. The unspecific adsorbtion of the FITC was determined by incubation of PMMA plates which were treated according to the protocol but lacking the hexamethylene diamine. PMMA plates initially coated with sandfish glycans were subsequently incubated with the FITC-NHS. The number of bound FITC molecules was determined by the reduction of fluorescence of the supernatant of the individual incubations. Fluorescence was determined using a plate reader (Glomax, Promega, Germany) with the FITC/Cy2 filter block.

### 2.7. Friction measurements

A simple, yet very effective method for evaluating the interaction between a surface and a particulate medium is friction measurement [4,8]. A sample surface was placed at a high inclination and sand was applied to it through a pipette, so it would slide off the surface. The inclination angle was then decreased until the flow stopped, at which point the angle (i.e. friction angle) was measured. From the angles, friction coefficients were calculated, which correlate by a tangent function.

### 2.8. Atomic force microscopy measurement

For atomic force microscopy (AFM) measurements, a Mobile S AFM (Nanosurf, Liestal, SUI) was used with a contact mode (CONTR) cantilever with a silicon tip in static force mode. Advancing and retracting force–distance curves were recorded and the adhesion peak difference was measured, which equals the adhesion force between the sample and the cantilever tip.

## 3. Results and discussion

It was obvious from lectin analysis [8] and from alternative techniques, like capillary electrophoresis [17], that a variety of different glycans are present in sandfish scales. To understand whether the complex mixture of different glycans is necessary as a whole to yield the friction reduction, or if only a subset of these molecules is necessary, glycans were prepared from scale lysates and separated by anion-exchange chromatography. Using a strong anion-exchange resin, neutral glycans were separated from possible acidic glycans and other negatively charged molecules. To test what influence the different glycans (or other molecules) have on surface interaction, they were covalently bound to PMMA by a newly developed protocol (see Material and methods). Three different samples were used in the modification protocol. The standard glycan sample containing all the structures, the neutral sample and the acidic sample. Afterwards sand friction angles were measured for the modified PMMA sheets.

**Figure 3.**
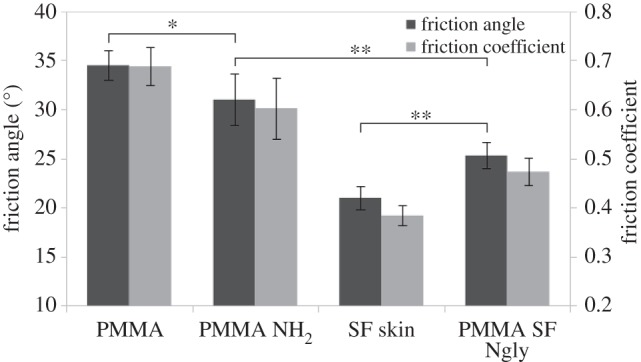
Friction measurements of sandfish epidermis and PMMA. Friction angle is shown on the left vertical axis for dark columns, while the calculated friction coefficient is shown on the right for bright columns. Ten independent measurements were performed for every sample. While there is a small but significant (*, confidence level *α* = 0.9, two sided unpaired *t*-test) reduction of friction due to activation of PMMA (PMMA-NH_2_) in comparison to untreated PMMA (PMMA), the further friction reduction due to glycosylation of PMMA (PMMA SF Ngly) is highly significant (**, *α* = 0.99). However, the level of the native sandfish scales (SF skin) is not fully reached using the current protocol.

Untreated PMMA exhibits a friction angle of 34.5 ± 1.51° which is similar to that of PMMA subjected to the identical activation treatment but without glycans (negative control) of 31.0 ± 2.62°. PMMA modified with the total glycan sample showed a significantly reduced friction with 25.3 ± 1.34°. Most interestingly the neutral glycan sample showed the same friction properties as the full sample 25 ± 1.21°, whereas the acidic samples showed a higher friction at 28.8 ± 1.62°. Thus, the neutral glycans can reduce the friction coefficient of PMMA by 35%, which is the reduction as can be obtained with the total mixture of sandfish glycans. This is summarized in figure 3.

**Figure 4.**
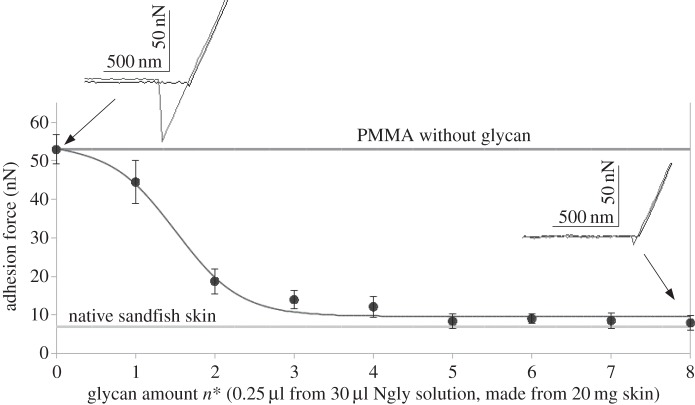
AFM adhesion force measurements of PMMA with different levels of glycosylation with neutral *N*-glycans. Typical force–distance curves are shown as insets for unglycosylated and ‘fully’ glycosylated PMMA. The adhesive force can be seen as height of the triangular snap in the retrace curve (fair curve). Clearly, a sigmoidal decrease of the adhesive forces can be observed with increasing glycosylation level. For comparison, the values of native sandfish skin and PMMA-NH_2_ are shown. Each data point represents the average unbinding force from 10 force–distance curves. Error bars depict the standard deviation.

As an alternative measure for quantifying the adhesion reduction, adhesive forces were measured with force–distance cycles employing AFM [5,8]. This technique also allows for fast and precise characterization of adhesion reduction with small samples, which is advantageous as the glycans are currently only available in small amounts. In figure 4, the dependence of the adhesion on the concentration of applied glycans is shown. Typical force–distance curves are depicted as insets. Clearly, PMMA in the absence of glycans exhibits large adhesive interactions, which can be identified as triangular peak in the curve displaying approximately 53 nN. With increasing glycan concentrations, the adhesive forces decrease and finally saturate at levels of about 10 nN which is similar to values observed on sandfish scales.

To determine the surface density of the glycans covalently bound to the PMMA surface, a fluorescence displacement assay was performed. For this, the NH_2_-bound fluorophores on PMMA in the presence and absence of bound *N*-glycans were quantified. The difference allowed us to estimate that at maximal coating density one glycan is bound on every 4 nm². This measurement is based on the assumption that the flurophores (fluorescein molecules) are much smaller than the glycans.

**Figure 5.**
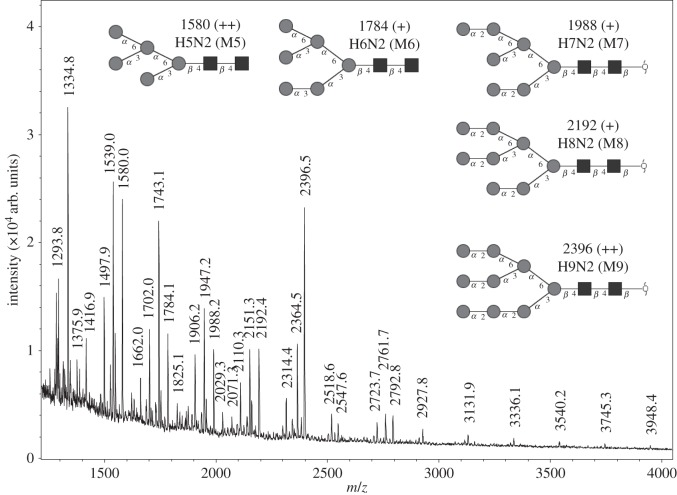
MALDI time-of-flight mass spectrometric analysis of *S. scincus* skin lysate *N*-glycans. Based on the masses and mass increments according to hexoses (204 *m*/*z*) and hexosamines (245 *m*/*z*), the inserted neutral glycans could be identified at the given *m/z*-values. Circles indicate mannose residues (Man), squares are hexosamines (GlcNAc). A semi-quantitative evaluation was made as follows: ++, major signal (more than 25% of the base peak intensity in the mass range *m/z* 1000–5000); +, significant signal with 5–25% of base peak intensity.

The data shown so far support the idea that the neutral glycans present in the scales play an important role in adhesion and friction reduction of surfaces. To identify the responsible glycans precisely, MALDI mass spectrometric profiling of enzymatically cleaved and permethylated *N*-glycans was performed. The profiles of sandfish β-keratins were characterized by a series of high-mannose *N*-glycans covering M5–M9 (figure 5). Most prominent were signals at *m*/*z* 1580 corresponding to Hex5HexNAc2 (M5) and at *m*/*z* 2396 corresponding to Hex9HexNAc2 (M9). The methylated glycans were registered as their sodium adducts (M + Na) and elimination products (M + Na − 32) or M + Na − 54). The structural assignments in the insets in figure 5 were corroborated by post-source decay analyses (MS2) to generate characteristic fragmentation spectra (not shown). The spectrum also indicated the presence of additional polyhexoses (series of signals with incremental mass differences of 204 (hexose increment) starting at *m/z* 1293).

The question arises as to whether the mannose-rich glycans found are sufficient to explain the adhesion and friction reduction. For this, alternative sources for the neutral glycans were searched for [18]. If neutral glycans from mistletoe (*Viscum album*) or from sweet almonds were purified and bound to PMMA by the same protocols as the neutral glycans from sandfish scales, the friction angles as well as the adhesion forces observed on these PMMA plates were indistinguishable from sandfish glycan-coated PMMA. If all glycans from an alternative source were used, the friction angle was even higher than for unglycosylated, yet aminated PMMA (31.2° ± 2.04°). The same holds true for the acidic sample (31.0° ± 2.36°). Only the neutral sample showed lower friction angles, almost exactly the values measured on the sandfish glycan-modified PMMA sheets, 25.4° ± 1.26°.

**Figure 6.**
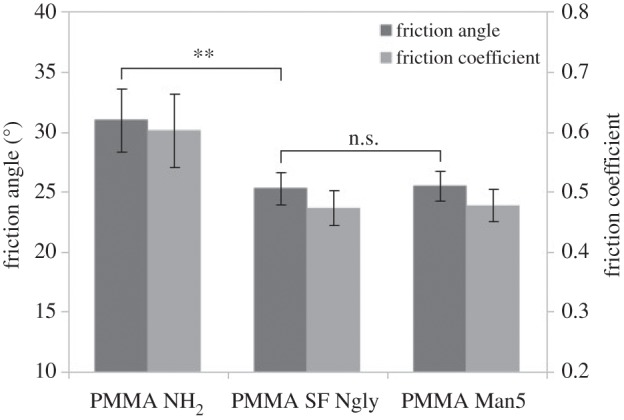
Friction measurements of modified PMMA with all sandfish glycans and with pure mannose 5 *N*-glycan (H5N2). The friction angles were measured 10 times on every sample. No significant difference between H5N2-coated PMMA and PMMA coated with all sandfish glycans can be detected (n.s., two sided *t*-test, *α* = 0.99) while there is a significant (**) reduction when compared to uncoated PMMA.

Now the question arises as to whether only one mannose-rich glycan can reduce friction like the full glycan sample from the sandfish. For this, a commercial purchased pure M5 (H5N2), containing two GlcNAcs and five mannoses, was used to coat PMMA. The data are shown in figure 6. This single glycan can reduce the friction by the same amount as the mixed *N*-glycans of the sandfish.

**Figure 7.**
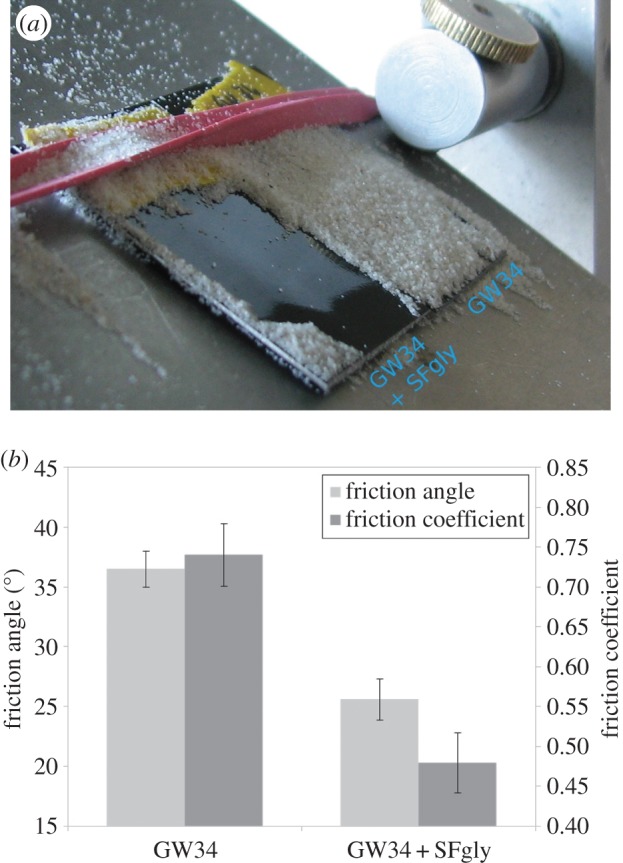
Sand adhesion and friction on native and glycosylated acrylic lacquer (brand name GW34). (*a*) A coated (GW34 + SFgly) and an untreated (GW34) sample were placed side by side on an inclined plane and completely covered with sand. The inclination was gradually increased until sand began to slide off the coated plate. The photograph was taken at an inclination of approximately 30°. (*b*) Direct comparison of friction angle and friction coefficient for native GW34 lacquer and glycosylated GW34. A friction reduction of approximately 40% could be achieved.

Finally, we asked ourselves whether the neutral glycans can also reduce the friction and adhesion of other, technically relevant polymers like acrylic lacquers. For this purpose, a commercially available car lacquer (GW34) was used and glycosylated/unglycosylated samples were compared with respect to sand friction. The results are shown in figure 7. While sand adheres on the untreated lacquer at an inclination angle of about 30°, it slides off the glycosylated surface. Most interestingly, the optical properties of the lacquer were not altered noticeably.

**Figure 8.**
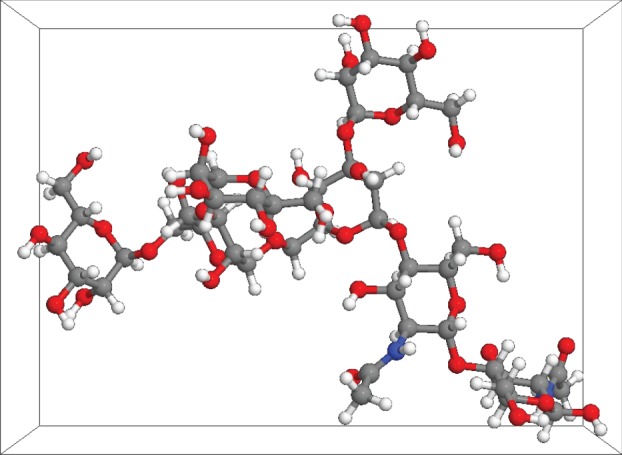
A three-dimensional model of a M5 (H5N2) glycan in a watery medium. The structure was obtained from GLYCAM database. All the atoms are labelled according to their nature. All the sugars contain carbon (grey), oxygen (red) and hydrogen (white) atoms, the GlcNAcs also contain nitrogen (blue). The cuboid around the molecule has dimensions of 2.25×1.65×1.24 nm.

Taken together, the results so far show that mannose-rich glycans can reduce van der Waals forces and thereby reduce adhesion and friction to polymeric surfaces. The exact molecular mechanisms remain to be determined. However, it is tempting to assume that the glycans act as molecular spacers. As the van der Waals potential decreases with distance to the power of 6, separating the interacting surfaces with a substance of low density. The rigid but branched glycans have less atoms per volume than the solid polymer. This can be estimated as follows. When the distance between surfaces, or particles in general, is very small they tend to attract each other due to van der Waals forces [19]. The affinity is pair specific and has a specific—the so-called Hamaker—interaction coefficient (*A*_Ham_) [19]. This is defined as the product of *π*, the interaction parameter *C* (particle–particle interaction) and the atom densities *ρ_n_* of both materials [20]:
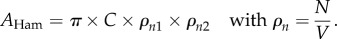


The number or particle density is a function of the crystal structure, and is defined as the number of atoms (*N*) in a physical volume (*V*). PMMA for example is a polymer of the monomer C_5_H_8_O_2_, which contains 15 atoms and has a molar mass of about 100 g mol^−1^ and the density of PMMA is 1190 kg m^−3^, leading to a number density of approximately 1.1 × 10^29^ atoms m^–3^ [21]. The number density of a Man5 glycan with a conformation as shown in figure 8 would probably differ from that of PMMA. Roughly approximated into a block (cuboid in figure 8) with the dimensions 2.25 × 1.65 × 1.24 nm, filled with 162 atoms, the glycan would have a particle density of approximately 35 × 10^27^ atoms m^–3^. Consider a flat PMMA slide, interacting with a spherical sand particle, composed of silicon dioxide. For such a simple system, there is an existing mathematical solution for the attractive van der Waals force [19], which is defined as follows:
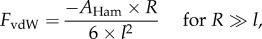
where *R* equals the particle radius, which is much greater than the distance to the PMMA surface *l*. There are no precise data available on particle–particle interaction, other than that of single elements [22,23] occurring between particles contained in molecules to date. However, assuming the slide is coated with Man5 glycans with a density of 35 × 10^27^ atoms m^–3^ in contrast to solid PMMA with 1.1 × 10^29^ atoms m^–3^, the van der Waals potential and thereby the van der Waals force is reduced. Focusing only on the difference in particle density, it would be by about a factor of 3 as A_Ham_ is linearly related to the density and the potential is linearly related to A_Ham_. Considering the data from [20,22], showing an attraction order Si > C > O > H, the difference may be even larger. Plentiful research is still required in this area, to create more precise mathematical models; however, adjusting particle density of an interface can considerably change surface interaction, especially adhesion, friction and concomitant abrasion.

The correlation of glycans, linked to the keratins and β-proteins, with reduced friction of the sandfish epidermis has been shown before [5,8]. This study set out to provide more details on the principle of friction reduction. We could provide detailed information on glycan structures which can reduce adhesion and friction of sand particles on sandfish scales as well as on technical surfaces. Demonstrations for industrial applications and a working model for the further research on friction reduction in general could be provided. If our hypothesis on ‘molecular spacers’ is confirmed, this will be a large step forward for friction related issues. These occur in all systems affiliated with motion—transportation, construction, process technology, etc.

## Supplementary Material

Response_Gly.pdf

## References

[1] Garsault FAP 1764 Les figures des plantes et animaux d'usage en medecine, d́ecrits dans la Matiere Medicale, p. 85. Paris: Geoffroy Medecin.

[2] Arnold EN, Leviton AE 1977 A revision of the lizard genus *Scincus* (Reptilia: Scincidae). Bull. Br. Mus. Nat. Hist. Zool. 31, 189–248.

[3] Hartmann UK 1989 Beitrag zur Biologie des Apothekerskinks *Scincus scincus**.* Herpetofauna 11, 17–39.

[4] Rechenberg I, El Khyari AR 2004 Reibung und Verschleiß am Sandfisch der Sahara. See http://www.bionik.tu-berlin.de/institut/festo04.pdf (accessed 15 January 2015).

[5] Baumgartner W, Saxe F, Weth A, Hajas D, Sigumonrong D, Emmerlich J, Singheiser M, Böhme W, Schneider JM 2007 The sandfish's skin: morphology, chemistry and reconstruction. J. Bionic Eng. 4, 1–9. (10.1016/S1672-6529(07)60006-7)

[6] Baumgartner W, Fidler F, Weth A, Habbecke M, Jakob P, Butenweg C, Böhme W 2008 Investigating the locomotion of the sandfish in desert sand by NMR-imaging. PLoS ONE 3, e3309 (10.1371/journal.pone.0003309) 18836551 PMC2561000

[7] Maladen RD, Ding Y, Li C, Goldman DI 2009 Undulatory swimming in sand: subsurface locomotion of the sandfish lizard. Science 325, 314–318. (10.1126/science.1172490) 19608917

[8] Alibardi L 2015 Immunolocalization of sulfhydryl oxidase in reptilian epidermis indicates that the enzyme participates mainly to the hardening process of the beta-corneous layer. Protoplasma 252, 1529–1536. (10.1007/s00709-015-0782-9) 25740419

[9] Staudt K, Böhme W, Baumgartner W 2012 Comparative investigations of the sandfish's β-keratin (Reptilia: Scincidae: *Scincus scincus*). Part 2: glycan-based friction reduction. J. Biomim. Biomater. Biomed. Eng. 16, 1–9. (10.4028/www.scientific.net/JBBTE.16.1)

[10] Staudt K, Saxe FPM, Schmied H, Soeur R, Böhme W, Baumgartner W 2012 Comparative investigations of the sandfish's β-keratin. Part 1: surface and molecular examinations. J. Biomim. Biomater. Biomed. Eng. 15, 1–16. (10.4028/www.scientific.net/JBBTE.15.1)

[11] Breloy I, Pacharra S, Ottis P, Bonar D, Grahn A, Hanisch F-G 2012 O-linked N,N’-diacetyllactosediamine (LacdiNAc)-modified glycans in extracellular matrix glycoproteins are specifically phosphorylated at subterminal N-acetylglucosamine. J. Biol. Chem. 287, 18 275–18 286. (10.1074/jbc.M111.280297) PMC3365741 22474328

[12] Kutz M 2002 Handbook of materials selection, p. 341. New York, NY: John Wiley & Sons.

[13] Meyers RA 1995 Molecular biology and biotechnology: a comprehensive desk reference, p. 722. Weinheim, Germany: Wiley-VCH.

[14] Brown L, Koerner T, Horton JH, Oleschuk RD 2006 Fabrication and characterization of poly (methylmethacrylate) microfluidic devices bonded using surface modifications and solvents. Lab Chip 6, 66–73. (10.1039/B512179E) 16372071

[15] Fixe F, Dufva M, Telleman P, Christensen CBV 2004 Functionalization of poly(methyl methacrylate) (PMMA) as a substrate for DNA microarrays. Nucleic Acids Res. 32, e9 (10.1093/nar/gng157) 14718554 PMC373302

[16] Bulmus V, Ayhan H, Piskin E 1997 Modified PMMA monosize microbeads for glucose oxidase immobilization. Chem. Eng. J. 65, 71–76. (10.1016/S1385-8947(96)03156-7)

[17] Vihar B 2015 Mimicking the abrasion resistant sandfish epidermis. PhD thesis, RWTH Aachen University, Germany.

[18] UniCarb Database. See http://unicarb-db.biomedicine.gu.se/ (accessed 19 March 2015).

[19] Parsegian VA 2005 Van der Waals forces: a handbook for biologists, chemists, engineers, and physicists, pp. 1–37. Cambridge, UK: Cambridge University Press.

[20] Israelachvili JN 2011 Intermolecular and surface forces, 3rd edn, pp. 415–467. Amsterdam, The Netherlands: Academic press.

[21] Buchholz K 2007 Plexiglas®. Werkstoff in Architektur und Design. Cologne, Germany: Wienand.

[22] Chu X, Dalgarno A 2004 Linear response time-dependent density functional theory for van der Waals coefficients. J. Chem. Phys. 121, 4083–4088. (10.1063/1.1779576) 15332953

[23] Tkatchenko A, Scheffer M 2009 Accurate molecular van der Waals interactions from groundstate electron density and free-atom reference data. Phys. Rev. Lett. 102, 0730051 (10.1103/PhysRevLett.102.073005) 19257665

