# Supplementary material for: Neutral glycans from sandfish skin can reduce friction of polymers
Source: J R Soc Interface. 2016 Mar;13(116):20160103. doi: 10.1098/rsif.2016.0103 (PMC4843684; doi:10.1098/rsif.2016.0103)
Supplement: Response_Gly.pdf [file rsif20160103supp1.pdf]

We would like to thank the referees for their positive comments and the constructive criticism which helped to improve the manuscript. Below please find a point by point reply to all remarks and criticisms.

Referee: 1

Abstract
….If these glycans ARE covalently bound…..
The sentence was changed as suggested.

---souses: SOURCES?
The typing error was corrected.

Introduction
-Pag 2, line 12:…..beta-keratins which are strongly glycosilated: NOTE, although it is not mandatory, the present authors may also considered that these non-keratin proteins are now termed Corneous Beta Proteins (see explanation in Alibardi, 2015, Protoplasma).
We now included the alternative nomenclature in the introduction and added the corresponding reference. However, as the term ß-keratin is very common and shorter, we sticked to this formulation throughout the rest of the manuscript.

-pag 2, line 14:…..der Waal forces: A REF WOULD USEFULF HERE
A reference to was added here.

-pag 2, line 18:….skinkid IS SCINCID
The mistake was corrected.

-pag 2, line 29:…..glycans can be DETECTED, compex……
The word was changed as suggested.

-PLEASE indicate better the goals of the study:…..what do you mean for “underlying effect”?
We tried to make the motivation and the goals of the study clear.

Materials and Methods
PLEASE USE THE TENSE consistently either Present (is, are) or Past-advised- (was, were)
The past-advise form is now used throughout the M&M-section.

-pag 2, line 43:….to OUR institute for…..
The typing error was corrected.

-page 3, line 29:…..PNGase…..: PLEASE spell it out bifore using the acronim and explain that this enzyme is needed to clave sugars from proteins (maybe providing a Reference)
We changed the sentence to:”To cleave the N-linked glycans from the peptide an aliquot of 0.5 µL Peptide -N-Glycosidase F, shortly named PNGase F (NEB, Frankfurt, Germany) was added and N-deglycosylation was performed at 37 °C for at least 16 h.”

-pag 3, line 41:….ones: please replace with GLYCANS.
The sentence was changed as suggested.

-pag 3. Line 48:……for modifications????:PLEASE be more specific, which modification/any or with coating procedures?
We now made clear that we mean covalently binding glycans to the surface.

-pag 4, line FITC: Please spell it out at first mention.
We spelled out Fluorescein isothiocyanate.

-pag 5, line 34:……to estimate then fully saturated…: THIS SENTENCE IS UNCLEAR????
Sorry, we changed the sentence to “The difference allowed to estimate that at maximal coating density one glycan is bound on every 4nm². ”

-pag 5, line 38:…..remove COMMA
The comma was removed.

-pag 5, line 50:….admisture????: UNCLEAR

We rewrote the sentence.

-pag 5, line54:…REMOVE COMMA
The comma was removed.

DISCUSSION
-on Pag 6 and 7 IS interesting. However, they do not discuss/interprete why one single mannone residue is as effective as a chain of mannoses, at least from my iiunderstanding. If I am correct why "nature" shouls have complicated its self with chains of mannoses or sugars?
Sorry for causing confusion, in fact no "single mannose residue" was used but a mannose rich glycan which is an N-glycan, containing 2 GlcNAcs and 5 mannoses, so perhaps 3-5 mannoses less, than the largest structures we found in the MS. Considering the synthesis on a molecular level, the glycans would be constructed by mannosyl-transferases, which link mannoses from GDP-mannoses (or similar) to an existing high-mannose glycan. It is questionable, how selective the enzyme is to the size of the glycan. As a result, it is likely, that the process would yield a spectrum of glycans with various sizes, depending on the environmental conditions.
We now tried to make this clear in the revised version by emhpasizing that a glycan and not a single residue was used.

-At the end of DISCUSSION a Conclusion of the findings of the present study on the general study on antifrictional materials should be presented. The authors should briefly discuss the importance of the present findings on the movevents of the sandfish and should better stress the potential knowledge or application IN GENERAL of studies on antifrictional properties.
A general conclusion was added as suggested.

Figure captions
-Fig. 1: ….during a short visit…: DO YOU MEAN "featured/photographed/observed during a short permanente on the sand surface"? …………not burrying themselves: DO YOU MEAN: that do not actively bury?......ARE mainly used to dig.
The paragraph was changed according to the suggestions.

-Fig. 2: is attached….by BONDING TO the methoxy groups….. For the present work…: DO YOU MEAN: in our method/procedure, the first……
The sentence was changed as suggested.

-Fig. 3: please report here the meaning of PMMA, PMMA NH2, SF skin, PMMA SF Ngly for an easier reading.
All abbreviations are now explained in the legend.

-Fig. 7. please report here the meaning of GW34 and GW34+SFgly for an easier reading.
The abbreviations in the figures are now explained in the legend.

-Fig. 8: the block around…: DO YOU MEAN the square/lines/geometric figure drawn around….
We wrote cuboid now for clarity.

Referee: 2

Comments to the Author

- On page 6 line 43 the authors compare number densities of glass with glycans. Since there is no friction data for glass in the paper a comparison between PMMA and glycans would be more consistent.
We now gave the comparison to PMMA as suggested.

- Instead of using an indirect approach to identify the surface coverage a direct measurement like i.e. XPS could be more convincing.
We completely agree that an indirect method is not as convincing as a direct approach. However, we spent some hard time in determining the surface density with different methods. We tried EDX and thaught of XPS but as we try to quantify glycans, which mainly consist of C, N and O (H can not be detected by any of these methods) on PMMA, which also consists of C, N and O, a reliable quantification is hardly possible.

- The equation on page 6 is unreadable in my copy of the manuscript.
Sorry for this, obviously during the pdf-conversion something happened. In the original word-file the formula is clear.
